# Heterogeneity and evolution of tumour immune microenvironment in metastatic gastroesophageal adenocarcinoma

**DOI:** 10.1007/s10120-022-01324-7

**Published:** 2022-07-29

**Authors:** Wei Wang, Liu-Fang Ye, Hua Bao, Ming-Tao Hu, Ming Han, Hai-Meng Tang, Chao Ren, Xue Wu, Yang Shao, Feng-Hua Wang, Zhi-Wei Zhou, Yu-Hong Li, Rui-Hua Xu, De-Shen Wang

**Affiliations:** 1 State Key Laboratory of Oncology in South China, Collaborative Innovation Center for Cancer Medicine, Sun Yat-Sen University Cancer Center, Sun Yat-Sen University, Guangzhou, 510060 People’s Republic of China; 2 Department of Gastric Surgery, Sun Yat-Sen University Cancer Center, Guangzhou, 510060 People’s Republic of China; 3 Research Unit of Precision Diagnosis and Treatment for Gastrointestinal Cancer, Chinese Academy of Medical Sciences, Guangzhou, 510060 People’s Republic of China; 4 Department of Medical Oncology, Sun Yat-Sen University Cancer Center, 651 Dong feng, East Road, Guangzhou, 510060 People’s Republic of China; 5 Geneseeq Research Institute, Nanjing Geneseeq Technology Inc., Nanjing, Jiangsu China; 6 School of Public Health, Nanjing Medical University, Nanjing, China

**Keywords:** Gastroesophageal adenocarcinoma, Tumour immune microenvironment, Spatial heterogeneity, TCR diversity, Immune cell infiltration

## Abstract

**Background:**

Tumour immune microenvironment heterogeneity is prevalent in numerous cancers and can negatively impact immunotherapy response. Immune heterogeneity and evolution in gastroesophageal adenocarcinoma (GEA) have not been studied in the past.

**Methods:**

Together with a multi-region sampling of normal, primary and metastatic tissues, we performed whole exome sequencing, TCR sequencing as well as immune cell infiltration estimation through deconvolution of gene expression signals.

**Results:**

We discovered high TCR repertoire and immune cell infiltration heterogeneity among metastatic sites, while they were homogeneous among primary and normal samples. Metastatic sites shared high levels of abundant TCR clonotypes with blood, indicating immune surveillance via blood. Metastatic sites also had low levels of tumour-eliminating immune cells and were undergoing heavy immunomodulation compared to normal and primary tumour tissues. There was co-evolution of neo-antigen and TCR repertoire, but only in patients with late diverging mutational evolution. Co-evolution of TCR repertoire and immune cell infiltration was seen in all except one patient.

**Conclusions:**

Our findings revealed immune heterogeneity and co-evolution in GEA, which may inform immunotherapy decision-making.

**Supplementary Information:**

The online version contains supplementary material available at 10.1007/s10120-022-01324-7.

## Introduction

Gastroesophageal adenocarcinoma (GEA) is an aggressive cancer that can metastasize to various sites [1]. Patients with metastatic GEA generally require systemic therapies. In recent years, a number of clinical trials have shown that immune checkpoint inhibitors (ICI) could improve the survival of metastatic GEA patients [2]. However, these studies have also found that ICI-responders constituted of only a small subset of GEA patients [2]. Tumour immune microenvironment (TIME) and its heterogeneity has been linked to ICI response in a number of cancers, however, the heterogeneity in TIME within primary tumours, and between normal gastric tissues, primary GEA and cancerous tissues at metastasis sites have not yet been studied.

The T cell receptor (TCR) repertoire and immune cell infiltration are two important aspects of the TIME. TCR repertoire represents the entire collection of TCR sequences and their abundances in an individual [3]. Several measures have been implemented to capture the different aspects of repertoire diversity such as TCR richness (number of unique TCR sequences), clonality (degree of even distribution), and Shannon entropy (accounting for both richness and clonality) [4]. TCR repertoire diversity measures are biologically important since they reflect the distribution of different T cell sub-populations and the evolutionary state of these sub-populations due to challenges from for example tumour neoantigen load [3]. These TCR diversity measures have clinical significance—shown to be predictive of immunotherapy response in a number of cancers [5–8]. However, investigation on TCR repertoire in GEA remains lacking.

Infiltration of various types of immune cells into the tumour microenvironment has been shown to play key role in tumour development. For example, neutrophils, macrophages and CD4 + regulatory T cells support tumour development, whereas CD8 + cytotoxic T cells, natural killer cells and gamma-delta T cells actively eliminate tumours [9]. Tumour immune cell infiltration has clinical significance in GEA. A meta-study found high infiltration of CD8 + T cells was associated with better overall survival [10]. Another recent study of metastatic GEA found lower CD8 + T cell level in metastasis compared to primary tumours [11].

These previous GEA studies, however, did not assess heterogeneity in TIME. TIME heterogeneity is prevalent in human cancers, and impacts both prognosis and immunotherapy response [12–18]. For example, patients with homogeneous T cell repertoire between tumour and adjacent normal lung had inferior survival, presumably due to a less tumour-focused T cell response [12].

Lack of immune heterogeneity studies in GEA stems from difficulty in acquiring specimens from multiple regions of normal tissue, primary tumour, and metastatic tissue from various sites from the same patient. However, we have access to a multi-region sampling of normal, primary, and metastatic tumour tissue, and conducted the first systematic analysis of immune heterogeneity and evolution in patients with metastatic GEA who underwent emergency surgery due to active tumour-related gastric bleeding and gastric antrum obstruction. We performed whole exome sequencing, TCR sequencing as well as immune cell infiltration estimation through deconvolution of gene expression signals, to examine the intra-tumour, intra-patient, and inter-patient tumour immune microenvironment heterogeneity in patients with GEA. Findings here may aid in the prediction of immunotherapy benefit and inform decision-making in the future.

## Methods and methods

### Patient recruitment and sample collection

Five patients with metastatic GEA who were initially admitted to the Department of Gastric Surgery (Sun Yat-sen University Cancer Center, Guangzhou, China), from November 2019 to July 2020, for emergency surgery due to active tumour-related gastric bleeding (*N* = 2) and gastric antrum obstruction (*N* = 3) were enrolled in this study. The patients’ primary gastric tumour tissues, adjacent normal gastric tissues and metastatic tumour tissue from lymph node, the peritoneum, liver and uterine attachment were retrieved at time of surgery. Tissue samples were immediately snap-frozen after resection or biopsy and stored in liquid nitrogen awaiting nucleic acid extraction. Two to three samples from normal and primary gastric tumour tissue were taken to assess spatial heterogeneity. Blood samples were collected before surgery from each patient. White blood was immediately transferred to cryogenic tubes for storage at − 80℃ awaiting germline DNA extraction.

### DNA extraction, library construction and whole exome sequencing

DNA from tissue samples and blood samples were extracted using a DNeasy Blood & Tissue Kit (Qiagen). Purified DNAs were quality checked using Nanodrop 2000 for A260/280 and A260/A230 ratios (Thermo Fisher Scientific). All DNA samples were quantified by Qubit 3.0 using the dsDNA HS Assay Kit (Life Technologies). Sequencing libraries were prepared using the KAPA Hyper Prep Kit (KAPA Biosystems). Target enrichment was performed using the xGen Exome Research Panel and Hybridization and Wash Reagents Kit (Integrated DNA Technology, USA) according to the manufacturer’s protocol. Libraries were subjected to PCR amplification with KAPA HiFi HotStart ReadyMix (KAPA Biosystems). The purified library was quantified using the KAPA Library Quantification Kit (KAPA Biosystems), and its fragment size distribution was analyzed using a Bioanalyzer 2100 (Agilent). Sequencing was performed on Illumina HiSeq4000 platform using PE150 sequencing chemistry (Illumina, USA) to a mean coverage depth of 150X for tissue samples, and 60X for matched normal control blood samples.

### Somatic mutation detection

FASTQ file quality control was performed using Trimmomatic [19], where N bases and low quality (score < 20) bases were removed. Pair-end reads were aligned to the human reference genome (hg19) using Burrows–Wheeler Aligner (BWA) with default parameters, followed by PCR deduplication with Picard V2.9.4 (Broad Institute, MA, USA). Local realignment around indels and base quality score recalibration was performed with the Genome Analysis Toolkit (GATK 3.4.0). Somatic single-nucleotide variants (SNVs) were identified using MuTect2. Final list of mutations was annotated using vcf2maf (available on GitHub). Resulting mutation list was filtered through an internal list. Somatic mutation calls were further filtered using the following criteria: (i) minimum 4 reads supporting the variant; (ii) ≥ 5% variant allele frequency (VAF); (iii) not present in public databases (Exome Variant Server, 1,000 genomes project and Exome Aggregation Consortium) at population frequency > 1%; (iv) in protein-coding regions.

### Analysis of metastatic seeding

Treeomics 1.7.3 [20], a well-established method specifically for multifocal tumours analysis for individual patients, was used to infer evolutionary relationships between primary tumour and metastasis. For each patient, high-confidence phylogeny rooted in the patient’s matched normal sample was derived based on SNVs/INDELs. Owing to the error-prone sequencing and varying neoplastic cell content, Treeomics implemented a Bayesian inference model to improve the accuracy in estimating the probability that a specific variant is present or absent. The global optimal tree was inferred based on Mixed Integer Linear programming. Phylogenetic tree consists of trunk mutations (those shared by all primary and metastatic samples), shared mutations (those shared by ≥ 2 samples) and private mutations (those found in only 1 sample).

### T cell receptor sequencing

DNA isolated from tissue and blood was subjected to T cell receptor (TCR) sequencing. In brief, the CDR3 region of TCR-beta chain was amplified with custom multiplex PCR primers (51 V-forward and 14 J-reverse primers) to capture all possible V(D)J combinations. Library was constructed and sequenced using the Illumina MiSeq platform to 10 M reads per sample. FASTQ file quality control was performed using Trimmomatic [19], where N bases and low quality (score < 20) bases were removed. MiXCR [21] was used to align and assign paired-end reads to CDR3 sequences, and clonotypes composed of CDR3 sequence and VDJ segment usage were quantified.

### TCR repertoire diversity analysis

Diversity measures assess the distribution of clonotype abundances within a sample’s TCR repertoire. We assessed 4 different diversity measures: richness, clonality, Shannon entropy and proportion of highly expanded clones. Richness represent number of unique clonotypes in a sample. Richness was normalized for read depth and relative T cell fraction (Supplemental Fig. S6) to allow fair comparison between samples. Clonality was defined as 1-Peilou’s evenness. High clonality (low evenness) implies skewing of clonotype abundance, whereas low clonality (high evenness) implies all clonotypes are nearly equally distributed. Shannon entropy is a measure that accounts for both richness and evenness [22]. Highly expanded clones (HEC) are defined as clonotypes with > 0.1% of total TCR repertoire in a sample.

### RNA extraction, library construction and whole transcriptome sequencing

Total RNA from tissue samples were extracted using an RNeasy Mini Kit (Qiagen). RNA purity was checked using Nanodrop 2000 for A260/280 and A260/A230 ratios (Thermo Fisher Scientific). All RNA samples were quantified by Qubit 3.0 using the RNA BR Assay Kit (Life Technologies). RNA integrity was assessed using Bioanalyzer 2100 (Agilent). RIN value (RNA integrity number) higher than 6.5 was required.

RNA sequencing libraries were prepared using KAPA Stranded RNA-Seq Kit with RiboErase (KAPA Biosystems). Briefly, rRNA was first depleted with RiboErase, followed by DNase digestion for DNA removal. Purified RNA was subjected to first strand cDNA synthesis, followed by second strand synthesis with dUTP marking for strand-specificity. This is followed by A-tailing, adapter ligation and library amplification. Final library was quantified using KAPA Library Quantification Kit (KAPA Biosystems), and its fragment size distribution was analyzed using a Bioanalyzer 2100 (Agilent). Sequencing was performed on the Illumina HiSeq4000 platform using PE150 sequencing chemistry (Illumina, USA) to an average of 60 M reads per sample.

### RNA sequencing data processing and transcript quantification

FASTQ file quality control was performed using Trimmomatic [19], where N bases and low quality (score < 15) bases were removed. Reads aligning to rRNA and tRNA sequences were removed. Cleaned reads were aligned to the human reference genome (hg19) using STAR v 2.5.2b [23], a splice-aware aligner. Transcripts were quantified using RSEM [24], which uses an expectation maximization algorithm to optimally assign reads that maps to multiple transcripts.

### Immune cell infiltration estimate

Relative infiltration level of 22 types of immune cell was estimated for each sample with CIBERSORT [25], using gene expression data (transcript per million) from RNA-seq. 22 types of immune cells include: B cell naïve, B cell memory, B cell plasma, T cell naïve, T cell CD4 + naïve, T cell CD4 + memory resting, T cell CD4 + memory activated, T cell CD8 + , T cell follicular helper, T cell regulatory, T cell gamma delta, macrophage M0, macrophage M1, macrophage M2, mast cell resting, mast cell activated, NK cell resting, NK cell activated, eosinophils, neutrophils, myeloid dendritic cell resting, myeloid dendritic cell activated. CIBERSORT package and gene expression signature matrix of 22 types of immune cells were downloaded from the web portal (http://cibersort.stanford.edu/) and ran locally. In brief, each sample has its gene expression profile. This profile is a linear combination of the known gene expression signature matrix of 22 types of immune cells and the relative fraction of those immune cells. CIBERSORT uses deconvolution through support vector regression to estimate the relative fraction of 22 types of immune cells.

### Immunomodulator gene expression analysis

Level of immunomodulation was assessed as per a previous study by Thorssen et al. Gene expression levels in transcript per million (TPM) of 70 immunomodulator genes were assessed [26]. These genes were divided into 7 super categories, including: receptor, ligand, co-stimulator, co-inhibitor, cell adhesion, antigen presentation and other. They can also be immune checkpoint inhibitors, stimulators or neither. Results were presented in a heatmap, where the median TPM of each gene was calculated for normal tissue, primary tumour and metastasis, and z-score normalized across the three tissue groups for each gene.

### Cytolytic activity

Cytolytic activity was calculated as the geometric mean of gene expression levels of GZMA and PRF1 as previously described [27].

### Neoantigen prediction

Neoantigen prediction is based on an assessment of the binding potential of mutation-derived peptide against patient’s MHC-I and MHC-II. HLA typing was performed using ATHLATES [28] and WES data. Neoantigens were predicted using pVAC-Seq [29] pipeline. Peptide substrings of 8 amino acids long containing mutant amino acid are identified and input into NetMHCPan v3 for binding affinity prediction. If any neopeptide substring exhibit IC50 binding affinity < 500 nM, the neopeptide is deemed to be an MHC binder and mutation is deemed a predicted neoantigen.

### Neoantigen and TCR repertoire and immune cell infiltration co-evolution analysis

Evolutionary relationships between primary and metastatic samples for each patient were constructed using hierarchical clustering based on Jaccard distance for neoantigen and TCR repertoire and Euclidean distance for immune cell infiltration. Co-evolution analysis involved the comparison of hierarchically clustered dendrogram trees between neoantigen, TCR repertoire and immune cell infiltration using cophenetic correlation from the dendextend package [30] as previously described [31]. Cophenetic correlation is the correlation between all pairwise distances in one dendrogram and the corresponding pairwise distances in the second dendrogram. Trees with cophenetic correlation *P* value < 0.05 was seen as having statistically significant co-evolution.

### Heterogeneity analysis

Several types of heterogeneity analysis were conducted. Pairwise comparisons were all made within each patient. Comparisons for all patients were represented with a boxplot. Intra-primary represents heterogeneity between different samplings from the same primary tumour. Inter-normal represents samples from spatially separated normal tissue within a patient. Inter-metastasis represents samples of spatially separated metastatic tissue. Three metastatic sites would mean 6 comparisons. Inter-primary-normal, inter-primary-metastasis, inter-metastasis-normal represent heterogeneity from the respective pairs of tissue categories. Euclidean distance was used to score the heterogeneity between samples. If two samples share a low amount of infiltrating immune cell abundance, the Euclidean distance would be greater, meaning that the two samples are more heterogeneous to each other.

### Immunotherapy biomarker and OncoKB actionable mutation analysis

Gene expression levels of well-established immunotherapy biomarkers including cytolytic activity (CYT), PD-L1 (PDCD1LG1), PD-L2 (PDCD1LG2), PD-1 (PDCD1), CD39, CD8A, CD8B and CD4 were z-score normalized across different samples within each patient. Tumour mutational burden (TMB) was counted by summing all non-synonymous alterations. CIS (chromosome instability score) was defined as the proportion of the genome with aberrant (purity-adjusted segment-level copy number ≥ 3 or ≤ 1) segmented copy number. OncoKB [32] v1.24 patch 1 was used to identify actionable mutations.

### Statistics

Non-parametric Wilcoxon’s rank-sum test was used to assess the differences between groups. *p* < 0.05 was considered to indicate a significant difference. False Discovery Rate was used for multiple testing correction. All statistical analyses were performed with R packages (v.3.5.3).

## Results

### Patient cohort overview

**Fig. 1:**
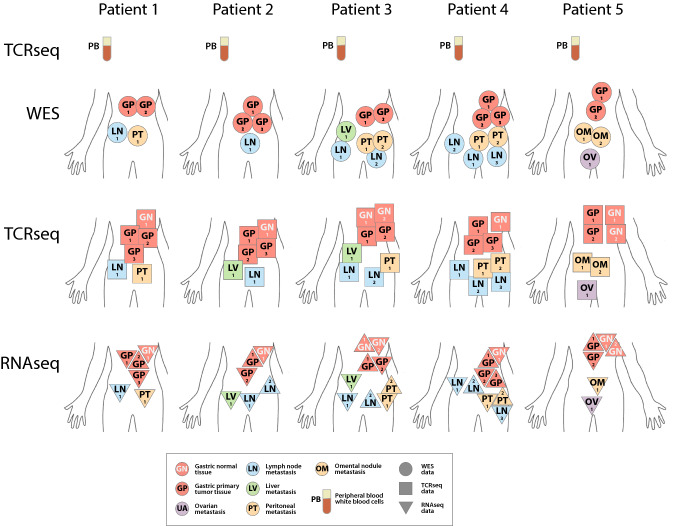
Patients, samples, and data description. Visual summary of where samples were obtained from for each patient

A total of 5 patients were enrolled in this study. All patients were treatment naïve prior to the surgery, of whom 2 males and 3 females. The median age of the patients was 53.4 (range, 39–67) years old. All patients had stage IV GEA, underwent palliative surgery due to tumour-related bleeding (*N* = 2) or gastric antrum obstruction (*N* = 3), had negative Epstein–Barr virus (EBV) status based on Epstein–Barr virus-encoded small RNAs (EBER) in situ hybridization, were microsatellite stable and did not have human leukocyte antigen loss of heterozygosity (HLA-LOH). Only one patient was HER2 positive (Table 1). The details of the biopsied regions for the five enrolled patients are shown in Fig. 1.

**Table 1:** Patient characteristics

Patient	Sex	Age	Locale	Stage	EBV	MSI	HLA-LOH
1	Male	59	Gastric antrum	T4bN3M1	–	Stable	–
2	Male	67	Gastroesophageal junction	T4aN3M1	–	Stable	–
3	Female	39	Gastric antrum	T4aN3M1	–	Stable	–
4	Female	61	Gastric body	T4aN3M1	–	Stable	–
5	Female	41	Gastric fundus	T4aN3M1	–	Stable	–

### T cell receptor repertoire diversity and heterogeneity

To explore the tumour immune microenvironment in metastatic GEA, we first assessed T cell receptor diversity and heterogeneity within and between normal, primary tumour and metastatic tissues.

**Fig. 2:**
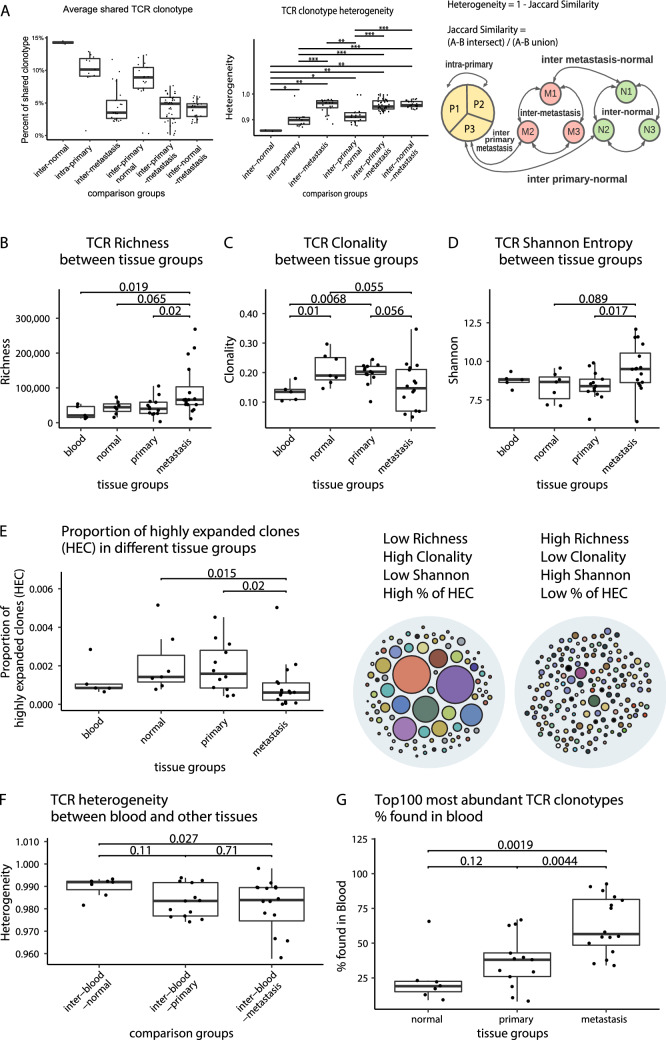
TCR repertoire diversity and heterogeneity within and across tissue groups. **A** TCR clonotype heterogeneity. Average percentage of shared TCR clonotypes among pairs are shown in the boxplot on the left. Heterogeneity is defined as one minus Jaccard similarity, where Jaccard similarity represents the degree of TCR clonotype sharing, which was calculated between each sample pairs within respective comparison groups. Only sample pairs within the same patient were compared. Schematic of comparison groups are depicted on the right side of (**A**). **B** TCR richness **C** TCR clonality and **D** TCR Shannon entropy between tissue groups. Richness is number of unique T cell receptor clonotypes; clonality is 1-Peilou’s evenness; Shannon entropy accounts for both richness and clonality. **E** Proportion of highly expanded clones (HEC) in different tissue groups. HEC are T cell receptor clonotypes accounting for > 0.1% of total TCR repertoire in a sample. **F** TCR heterogeneity between blood and other tissues. Heterogeneity and comparisons are as described in (**A**). **G** Top100 most abundant TCR clonotypes % found in blood. For (**B**), (**C**), (**D**), (**E**), (**F**) and (**G**), 2-sided Wilcoxon test was used *P ≤ 0.05, **P ≤ 0.01, ***P ≤ 0.001 (only significant or trend toward significance results are shown)

In terms of TCR heterogeneity, it was highest amongst metastatic samples (inter-metastasis), with an average only 4.67% of TCR clonotypes were shared, as well as between primary tumour and metastatic samples (inter-primary-metastasis) with 4.26% shared, and between normal gastric tissue and metastatic samples (inter-normal-metastasis) with 3.99% shared (Fig. 2A). TCR heterogeneity was lower among primary samples (intra-primary) (Fig. 2A), with on average 10% of TCR clonotypes shared. A total of 113 pairwise comparisons (inter-normal: *N* = 2; intra-primary: *N* = 9; inter-metastasis: *N* = 21; inter-primary-normal: *N* = 17; inter-primary-metastasis: *N* = 41; inter-normal-metastasis: *N* = 23) were made for heterogeneity analysis. A schematic of how the comparisons were made is shown in Fig. 2A.

Next, we assessed the diversity of T cell receptor clonotype among normal, primary tumour and metastasis. Compared to the primary tumour and normal gastric tissue, metastatic samples had higher TCR richness (number of unique TCR clonotypes) (Fig. 2B) (on average 92,968, 45,048, 43,803 and 29,812 unique TCR clonotypes detected in metastatic, primary, normal and blood, respectively). To ensure a fair comparison between samples, we supplemented this analysis with T cell proportion data. From the CIBERSORT results, we isolated 7 infiltrating T cell fractions. Then, we normalized TCR richness by the T cell proportion in each tissue type. Supplemental Fig. S6 shows the results, which indicated that normalizing by infiltrating T cell fraction did not drastically alter the relative TCR richness for each tissue type. Metastatic samples also had lower clonality (Fig. 2C) and higher Shannon’s entropy (Fig. 2D). Together with the lower proportion of highly expanded clones (Fig. 2E), low clonality suggests very few T cell clones are expanded and active in metastatic samples. In some patients, even within normal, primary, or metastatic tissue groups, there were differing levels of TCR diversity. For example, in patient 3, two primary tumour samples have dramatic differences in TCR richness as well as clonality (Supplemental Fig. S1).

We further divided the metastasis group into the lymph node and other metastasis. The purpose of this was to determine how lymph nodes, which filter T cells regularly, may present antigen in a different manner before and after developing metastasis. We found that the concordance of the TCR repertoire of lymph node metastasis and the primary tumour is lower than other metastases and primary tumour (Supplemental Fig. S7). Based on this, we deduce that this is due to the circulating T cells being captured during sample collection. In this case, a portion of unrelated TCRs would be captured, thus resulting in the observed diverse repertoire.

To gauge the amount of circulatory immune surveillance in metastatic GEA, we assessed TCR clonotype sharing between blood and normal tissue, primary tumour and metastasis. There is very little TCR clonotype sharing between blood and normal, primary or metastatic tissue (Fig. 2F). Only ~ 1% is shared between blood and normal tissue, and ~ 2% is shared between blood and primary or metastatic tissue. Low degree of TCR clonotype sharing between blood and tumour tissues was also found in other cancers (Cowell,2020). However, looking at the top 100 most abundant TCR clones in different tissue samples, metastasis shares 56.5% with blood (Fig. 2G), whereas only 38% and 19% was shared between blood and primary tumour and normal tissue respectively.

We also assessed T cell receptor’s VJ segment differential usage between blood, normal gastric tissue, primary tumour and metastatic tissue. Highest number of differential VJ usage was found between blood and the other tissue types (Supplemental Fig. S2A, B, F). This is expected since we saw very little TCR clonotype sharing between blood and other tissue types. Normal gastric tissue was also enriched in many VJ segments not seen in metastatic tissue (Supplemental Fig. S2E). Most interestingly, one VJ segment (TRBV25-1 TRBJ2-5) was enriched in the primary tumour compared to metastatic tissue (Supplemental Fig. S2D).

Together these results suggest a highly heterogeneous T cell population among metastatic sites. T cells are being supplied to metastatic sites via blood. However, these T cell clones are not yet expanded and not active in metastasis.

### Immune cell infiltration and immunomodulation

**Fig. 3:**
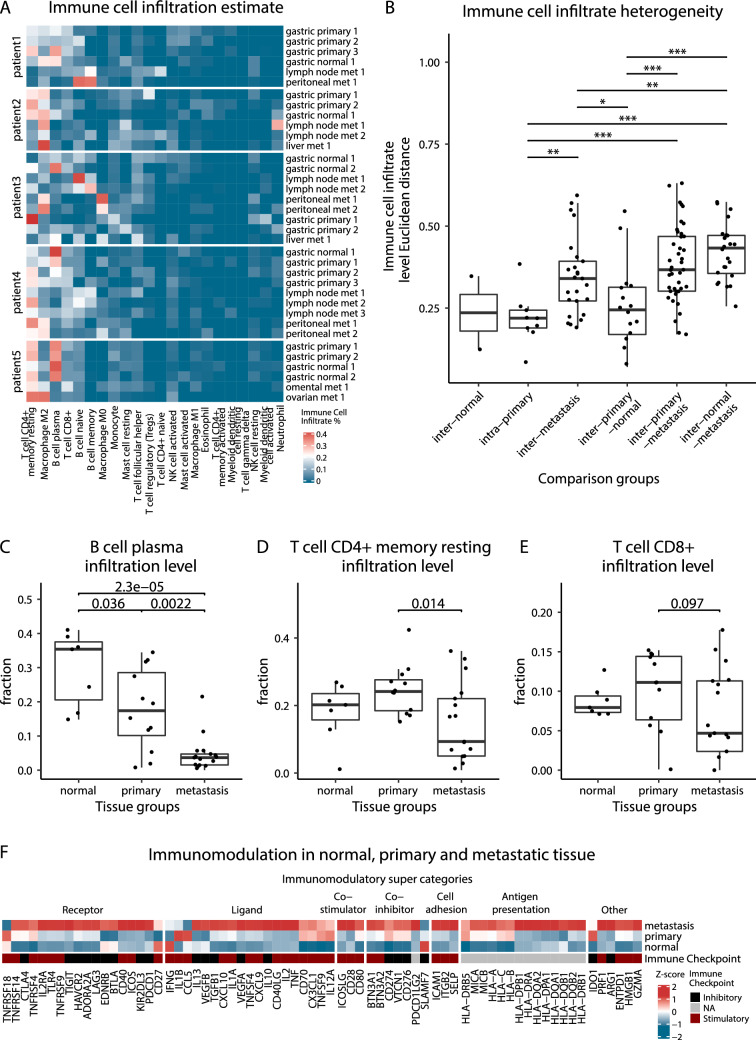
Immune microenvironment status within and across tissue groups. **A** Immune cell infiltration estimate. Infiltration % of 22 types of immune cell was estimated for each sample with CIBERSORT using gene expression data from RNA-seq. **B** Immune cell infiltration heterogeneity. Immune cell infiltration divergence is calculated as the Euclidean distance between immune cell infiltrate % of a pair of samples; Only sample pairs within same patients were compared; 2-sided Wilcoxon test was used. *P ≤ 0.05, **P ≤ 0.01, ***P ≤ 0.001 (only significant or trend toward significance results are shown). **C** B cell plasma **D** T cell CD4 + memory resting **E** T cell CD8 + infiltration levels. 2-sided Wilcoxon test was used (only significant or trend toward significance results are shown). **F** Immunomodulation in normal, primary and metastatic tissue. Immunomodulation is characterized using gene expression level of 70 genes involved in immunomodulation (Thorsson, 2018) [26]. Heatmap TPM (transcript per million) value is the median of all samples from respective tissue groups, z-score normalized across the 3 tissue groups

Next, we assessed immune cell infiltration levels and heterogeneity within and between normal, primary tumour and metastatic tissues using CIBERSORT [25], which quantified the relative abundance of 22 types of immune cells within a sample. Some of these immune cell types such as CD8 + T cells, CD4 + T cells and plasma cells are directly involved in tumour immune response [9, 33, 34]. Among 22 immune cells, infiltration level is highly heterogeneous among tissue samples (Fig. 3A). Similar to TCR repertoire, heterogeneity of immune cell infiltration levels was highest amongst metastatic samples (inter-metastasis), between primary and metastatic samples (inter-primary-metastasis) and between normal gastric tissue and metastatic samples (inter-normal-metastasis) (Fig. 3B). Immune cell infiltration heterogeneity was lower among primary samples (intra-primary) and among normal samples (inter-normal) (Fig. 3B). Infiltration of immune cells involved in tumour elimination (CD8 + T cells, CD4 + T cells and plasma cells) were significantly lower in metastasis compared to primary tumour (Fig. 3C–E). This confirms results from another recent study which also found metastasis to have lower CD8 + T cell infiltration compared to primary tumour in GEA (11). (For all 22 immune cell infiltration comparisons among normal, primary and metastatic tissues, see Supplemental Fig. S3A–W).

We also assessed immunomodulation via the expression of 70 immunomodulatory genes as previous described [26]. On average, metastatic samples were undergoing heavy immunomodulation compared to a quieter immunomodulatory environment of the primary tumour and normal tissue despite showing less immune cell infiltration of CD4 + T cells, CD8 + T cells, and plasma cells (Fig. 3F). However, as is shown in Supplemental Fig ure S3A–W, these three cell types make up only a fraction of the immune cell environment as predicted by CIBERSORT. In fact, almost half of the immune infiltrating cell types included in the analysis show higher infiltration in metastases than either normal or primary samples. Among these are B cell naive (Supplemental Fig. S3A), B cell memory (Supplemental Fig. S3B), Macrophage M0 (Supplemental Fig. S3N), and Macrophage M2 (Supplemental Fig. S3P). Furthermore, as transcription of antigen-presenting genes is increased in metastasis, immune checkpoint stimulatory genes are enhanced as well. They regulate and hinder the infiltration of T cells, which results in the lower fractions of T cells found in metastasis samples. (For immunomodulatory status of each sample, see Supplemental Fig. S3X).

Together these results show a highly heterogeneous immune cell infiltration landscape among metastases. Lower infiltration of tumour eliminating immune cells in metastatic tissue, and a heavily immunomodulated metastatic environment.

### Neoantigen, TCR repertoire and immune cell infiltration co-evolution

**Fig. 4:**
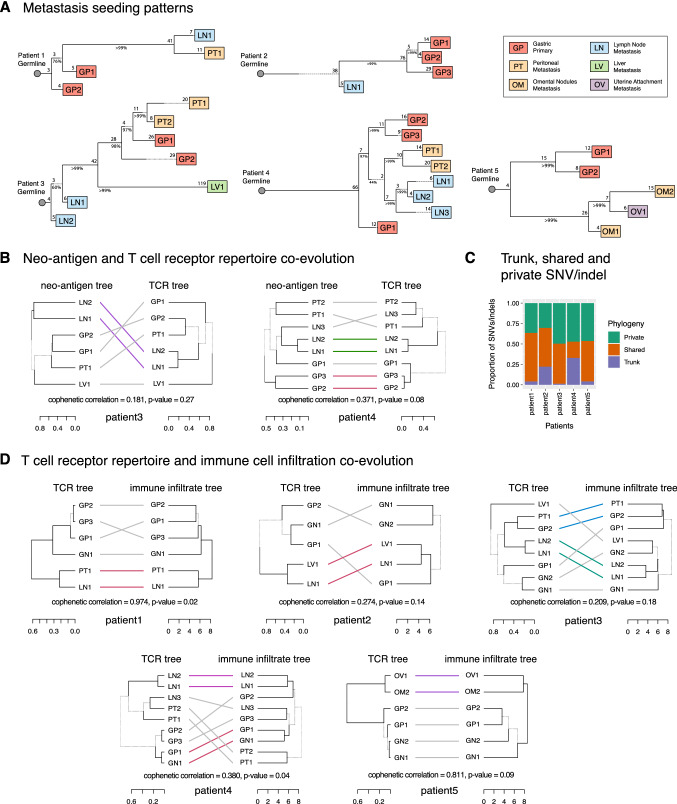
Mutational, neo-antigen, TCR and immune cell infiltration evolution. **A** Metastasis seeding patterns. Metastasis seeding patterns were constructed with Treeomics using whole exome sequencing data. Grey circles represent germline state of each patient. Length of tree branches are proportional to the number of mutations prior to branching or termination at leaf nodes, which is indicated at respective branch points or leaf nodes. **B** Neo-antigen and T cell receptor co-evolution. Neo-antigen and TCR trees were constructed using hierarchical clustering in complete mode. Trees were compared using cophenetic correlation. **C** Trunk, shared and private SNV/indel. In each patient, trunk SNV/indels are those shared by all samples. Shared SNV/indels are those shared by two or more samples. Private SNV/indels are those found in only one sample. **D** T cell receptor repertoire and immune cell infiltration coevolution. TCR and immune cell infiltration trees were constructed using hierarchical clustering in complete mode. Trees were compared using cophenetic correlation

Next, we wanted to see whether if mutational profile, TCR repertoire and immune cell infiltration evolved along similar paths. Interestingly, we found patients with a large proportion of trunk mutations and a low level of mutational heterogeneity among samples (patient 4, Fig. 4C, Supplemental Fig. S4A) had a high and significant correlation between the neo-antigen tree and TCR repertoire tree [correlation = 0.371, *P* value (0.08)] (Fig. 4B). This suggests that co-evolution between neo-antigen and TCR repertoire may be correlated to patients with a late divergent mutational evolution. In contrast, those with very few trunk mutations and a high level of mutational heterogeneity among samples (patient 3, Fig. 4C, Supplemental Fig. S4A) had a low and non-significant correlation between neo-antigen tree and TCR repertoire tree [correlation = 0.096, *P* value (0.33)] (Fig. 4B). This indicates in patients with early divergent mutational evolution, there is no co-evolution of neo-antigen and TCR repertoire. (For comparisons of neo-antigen and TCR repertoire trees of all patients, see Supplemental Fig. S4B–F).

There appears to be co-evolution of TCR repertoire and immune cell infiltration among most patients (except patient 2) (Fig. 4D). This indicates the possibility that immune cell infiltration is acting in concert with T cell expansion.

### Heterogeneity in immunotherapy biomarkers

**Fig. 5:**
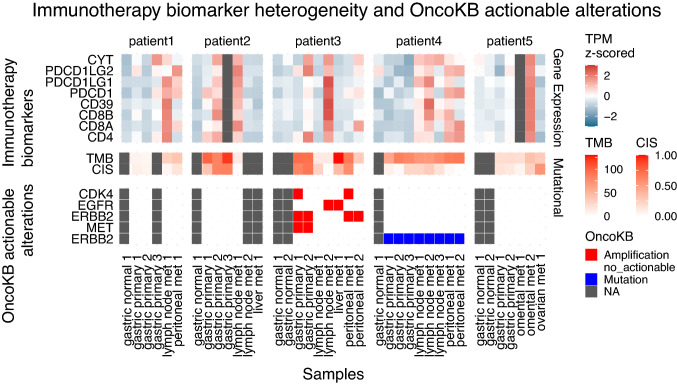
Immunotherapy biomarker heterogeneity and OncoKB actionable alterations. Top: gene expression of immunotherapy biomarkers is expressed in TPM (transcript per million), and z-score normalized across different samples in each patient. Middle: TMB (tumour mutational burden) was calculated as the total number of non-synonymous mutations in a sample. CIS (chromosome instability score) was defined as the proportion of the genome with aberrant (purity-adjusted segment-level copy number ≥ 3 or ≤ 1) segmented copy number. Bottom: OncoKB actionable variants v1.24 patch 1 was used. Grey squares show not applicable or inadequate samples

Lastly, we assessed expression levels of well-known immunotherapy biomarkers across samples. We found these immunotherapy biomarkers are heterogeneous across samples (Fig. 5). This spatial heterogeneity of immunotherapy biomarkers points to the inadequacy of using biomarker results from a single sample for clinical decision making. Most patients lack actionable OncoKB alterations. OncoKB gene amplifications appear to be heterogeneous in patient 3, whereas OncoKB ERBB2 mutation was homogeneous in patient 4.

## Discussion

Heterogeneity of tumour immune microenvironment complicates the assessment of immunotherapy biomarkers and can lead to non-uniform response to immune checkpoint inhibitors (ICIs). In this study, we performed multi-region sampling of normal gastric tissue, primary tumour and metastatic tissue from 5 treatment naïve patients. Using a multi-omics approach, we analyzed T cell receptor repertoire and immune cell infiltration, along with their heterogeneities both within and between the tissue samples. We also compared evolutionary trajectories between the tumour genome and immune microenvironment.

We found that, when compared to normal and primary gastric tumours, metastasis in GEA had a pro-tumour immune microenvironment. Metastasis had high richness but low TCR clonality, which represents a lack of dominant and potentially tumour-targeting T cell clones (Supplemental Fig. S5). Previous studies have found those with high TCR clonality prior to anti-PD-1 therapy had longer progression-free survival in metastatic melanoma [35], and better response to immunotherapy in malignant pleural mesothelioma [36]. There was a high degree of overlap of major TCR clonotypes between metastatic TCR repertoire and circulating TCR repertoire found in blood, which indicates a high level of immune surveillance from blood. However, given low clonality in metastasis, it would appear these T cells are not yet active or may be suppressed by the metastatic tumour microenvironment. Metastasis also had lower infiltration of tumour-eliminating immune cells, including CD8 + T cells, CD4 + memory resting T cells, and plasma cells. Plasma cells appear to form tertiary lymphoid structures with CD8 + and CD4 + T cells in ovarian cancer, where the prognostic benefit is restricted to tumour that have both high CD8 + , CD4 + T cells and plasma cell infiltrations [37]. Another study also found CD138 + plasma cells were associated with non-small cell lung cancer (NSCLC) prognosis [38].

For both T cell receptor repertoire and immune cell infiltration, there was high heterogeneity amongst metastatic samples compared to the relatively homogeneous immune landscape of primary tumour and normal gastric samples (Supplemental Fig. S5). Same metastatic organs also exhibited drastically different levels of immune cell infiltration, as well as TCR diversity measures. Diverse levels of immune cell infiltration across metastases were also seen in breast cancer [31]. The diverse immune landscape among metastasis may explain the heterogeneous fate of lesions following treatment. A seminal case study of an ovarian cancer patient recently demonstrated while some metastatic lesions regressed, others progressed. This was reflected by immune cell infiltration versus exclusion respectively [39]. Future longitudinal studies are needed to assess whether this is the case in metastatic GEA.

To gauge the interaction between tumour genome changes and response of the host immunity, we compared the evolution of neo-antigen, TCR repertoire and immune cell infiltration. Neo-antigens are mutant peptides capable of triggering T cell clonal expansion [40]. We found in patients with early divergent mutational evolution and high mutational heterogeneity, there was a lack of co-evolution of neo-antigen and TCR repertoire; whereas if a patient has late divergent mutational evolution and low mutational heterogeneity, a co-evolution of neo-antigen and TCR repertoire can be seen. This late divergent mutation pattern was observed in patients 2 and 4 and could be a result of late metastatic seeding. It is likely that because the metastasis arose recently and was seeded directly from the primary tumour, there was not sufficient time for divergence in TCR repertoire or neo-antigen repertoire to occur between the samples. On the other hand, the connection between TCR repertoire and immune infiltrate evolution likely originates from the array of antigen being presented from the tumour site. The specificity of binding of antigen to TCR means that passing T cells which recognize and bind to the antigen will localize. These activated T cells will clone itself and recruit fellow immune cells for assistance. However, given the limited number of patients in this study, these findings remain speculative. A larger cohort is needed to confirm these findings.

Finally, we found the expression of immunotherapy biomarkers, as well as TMB and CIS, were heterogeneous. Similar region-to-region heterogeneity has also been observed for PD-1 and PD-L1 expression in NSCLC [41]. This heterogeneity in GEA may explain the difficulty in finding a robust immunotherapy biomarker and heterogeneous responses to immunotherapy.

Using a multi-region, multi-omics approach, this study revealed a heterogeneous immune landscape in metastatic GEA with mutational-immune co-evolution in certain patients. These findings may have implications for immunotherapy decision-making.

## Supplementary Information

Below is the link to the electronic supplementary material.

Supplementary file1 (PDF 5872 KB)
