# Supplementary material for: Heterogeneity and evolution of tumour immune microenvironment in metastatic gastroesophageal adenocarcinoma
Source: Gastric Cancer. 2022 Jul 29;25(6):1017–30. doi: 10.1007/s10120-022-01324-7 (PMC9587966; doi:10.1007/s10120-022-01324-7)
Supplement: Supplementary file 1 — Supplementary file1 (PDF 5872 KB) [file 10120_2022_1324_MOESM1_ESM.pdf]

Article title:

Heterogeneity and evolution of tumour immune microenvironment in metastatic gastroesophageal adenocarcinoma

Journal title: Gastric Cancer

Authors:
Wei Wang[1,2¶], Liu-Fang Ye[1,3,4¶], Hua Bao[5¶], Ming-Tao Hu[1,3,4], Ming Han[5], Hai-Meng Tang[5], Chao Ren[1,3,4], Xue Wu[5], Yang Shao[5,6], Feng-Hua Wang[1,3,4], Zhi-Wei Zhou[1,2], Yu-Hong Li[1,3,4], Rui-Hua Xu[1,3,4], De-Shen Wang[1,3,4]*

Affiliations:
[1] State Key Laboratory of Oncology in South China, Collaborative Innovation Center for Cancer Medicine, Sun Yat-sen University Cancer Center, Sun Yat-sen University, Guangzhou 510060, P. R. China;
[2] Department of Gastric Surgery, Sun Yat-sen University Cancer Center, Guangzhou, 510060, P.R. China;
[3] Research Unit of Precision Diagnosis and Treatment for Gastrointestinal Cancer, Chinese Academy of Medical Sciences, Guangzhou 510060, P. R. China;
[4] Department of Medical Oncology, Sun Yat-sen University Cancer Center, Guangzhou, 510060, P.R. China;
[5] Geneseeq Research Institute, Nanjing Geneseeq Technology Inc. Nanjing, Jiangsu, China;
[6] School of Public Health, Nanjing Medical University, Nanjing, China;
[¶] These authors contributed equally to this study

Corresponding Author:
*De-Shen Wang, MD, PhD
Department of Medical Oncology, Sun Yat-Sen University Cancer Center, State Key Laboratory of Oncology in South China, Collaborative Innovation Center of Cancer Medicine, 651 Dong feng East Road, Guangzhou 510060, China.
Email: wangdsh@sysucc.org.cn.
Telephone: +86-20-8734 3351, Fax: +86-20-87343351.

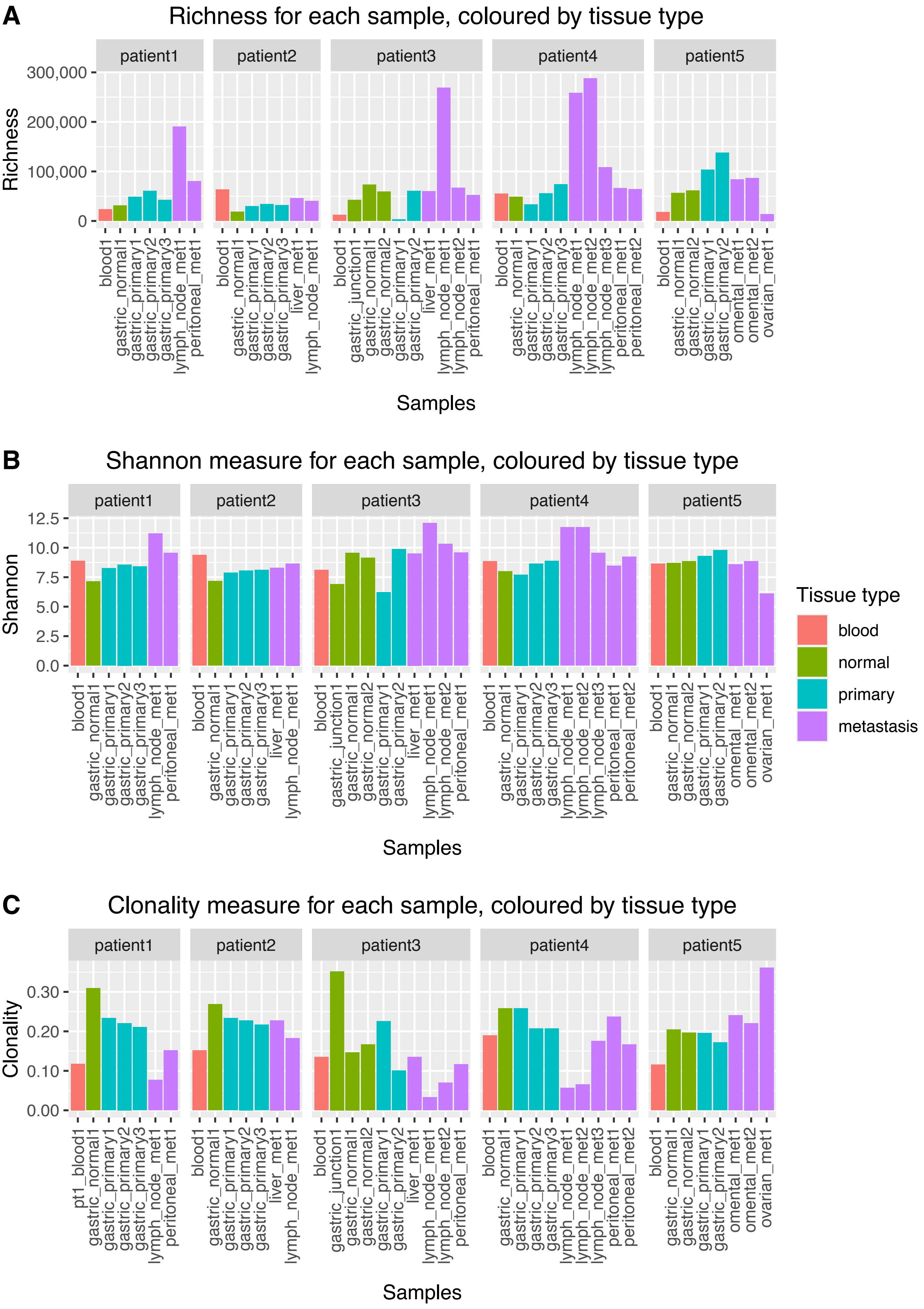
A
Richness for each sample, coloured by tissue type
patient1
patient2
patient3
patient4
patient5
Richness
300,000
200,000
100,000
0
blood1
gastric_normal1
gastric_primary1
gastric_primary2
gastric_primary3
lymph_node_met1
peritoneal_met1
liver_met1
gastric_junction1
gastric_normal2
lymph_node_met2
lymph_node_met3
peritoneal_met2
omental_met1
omental_met2
ovarian_met1
Samples
B
Shannon measure for each sample, coloured by tissue type
Shannon
12.5
10.0
7.5
5.0
2.5
0.0
Samples
Tissue type
blood
normal
primary
metastasis
C
Clonality measure for each sample, coloured by tissue type
Clonality
0.30
0.20
0.10
0.00
pt1_blood1
Samples

Supplemental Figure S1 TCR diversity measures across samples (A) TCR richness across samples. Richness is number of unique T cell receptor clonotypes. (B) TCR clonality across samples. Clonality is calculated as 1-Peilou's evenness, where Peilou's evenness equals Shannon entropy normalized by ln(total number of species). (C) TCR Shannon entropy across samples. Shannon entropy is a diversity measure that accounts for both richness and clonality. Samples are coloured according to their tissue type.

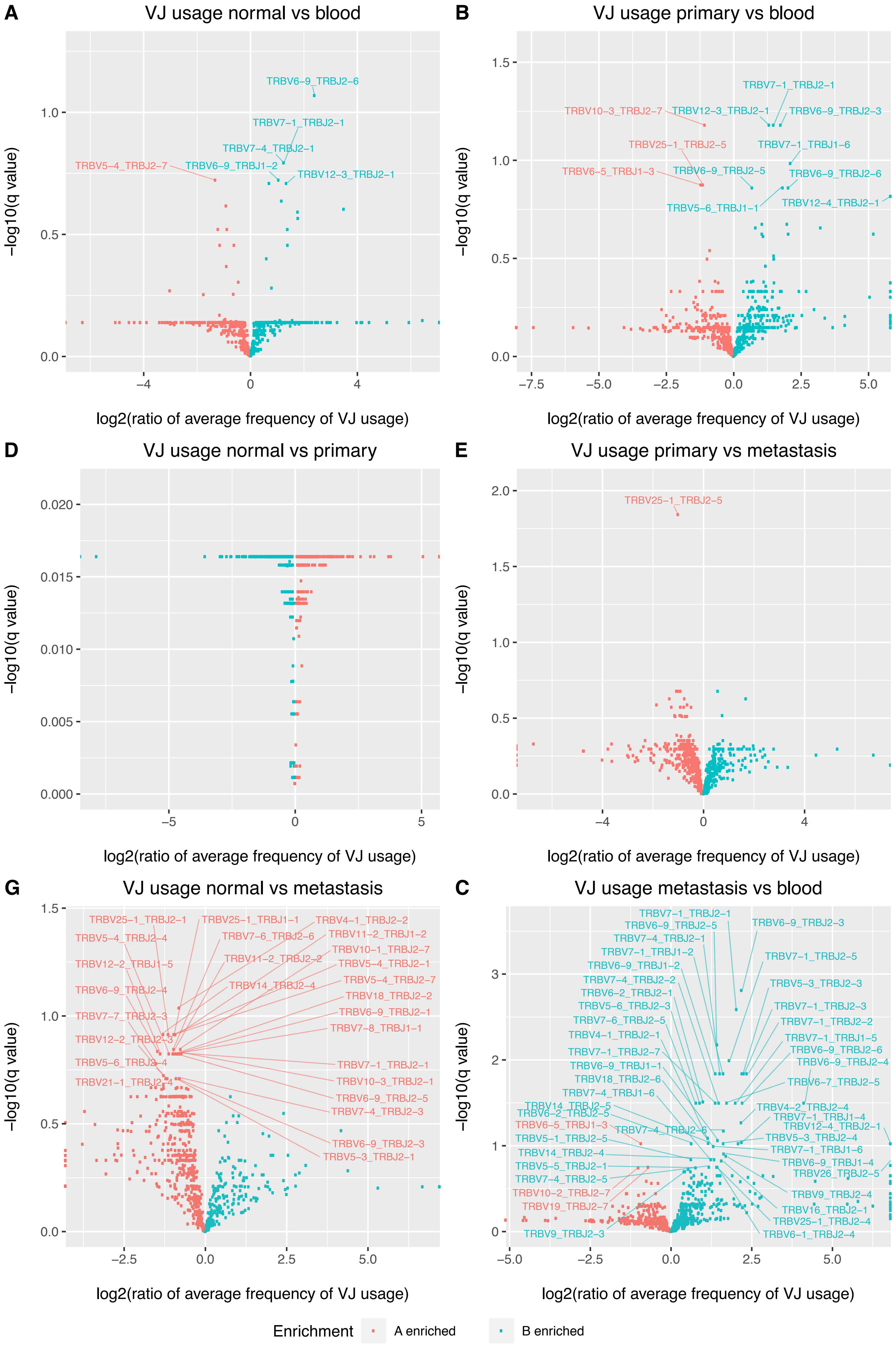

A
VJ usage normal vs blood
TRBV6−9_TRBJ2−6
TRBV7−1_TRBJ2−1
TRBV7−4_TRBJ2−1
TRBV5−4_TRBJ2−7
TRBV6−9_TRBJ1−2
TRBV12−3_TRBJ2−1
1.0
0.5
0.0
−4
0
4
−log10(q value)
log2(ratio of average frequency of VJ usage)
B
VJ usage primary vs blood
TRBV7−1_TRBJ2−1
TRBV10−3_TRBJ2−7
TRBV12−3_TRBJ2−1
TRBV6−9_TRBJ2−3
TRBV25−1_TRBJ2−5
TRBV7−1_TRBJ1−6
TRBV6−5_TRBJ1−3
TRBV6−9_TRBJ2−5
TRBV6−9_TRBJ2−6
TRBV5−6_TRBJ1−1
TRBV12−4_TRBJ2−1
1.5
1.0
0.5
0.0
−7.5
−5.0
−2.5
0.0
2.5
5.0
−log10(q value)
log2(ratio of average frequency of VJ usage)
D
VJ usage normal vs primary
0.020
0.015
0.010
0.005
0.000
−5
0
5
−log10(q value)
log2(ratio of average frequency of VJ usage)
E
VJ usage primary vs metastasis
TRBV25−1_TRBJ2−5
2.0
1.5
1.0
0.5
0.0
−4
0
4
−log10(q value)
log2(ratio of average frequency of VJ usage)
G
VJ usage normal vs metastasis
TRBV25−1_TRBJ2−1
TRBV25−1_TRBJ1−1
TRBV4−1_TRBJ2−2
TRBV5−4_TRBJ2−4
TRBV7−6_TRBJ2−6
TRBV11−2_TRBJ1−2
TRBV10−1_TRBJ2−7
TRBV12−2_TRBJ1−5
TRBV11−2_TRBJ2−2
TRBV5−4_TRBJ2−1
TRBV5−4_TRBJ2−7
TRBV6−9_TRBJ2−4
TRBV14_TRBJ2−4
TRBV18_TRBJ2−2
TRBV7−7_TRBJ2−3
TRBV6−9_TRBJ2−1
TRBV7−8_TRBJ1−1
TRBV12−2_TRBJ2−3
TRBV5−6_TRBJ2−4
TRBV7−1_TRBJ2−1
TRBV21−1_TRBJ2−4
TRBV10−3_TRBJ2−1
TRBV6−9_TRBJ2−5
TRBV7−4_TRBJ2−3
TRBV6−9_TRBJ2−3
TRBV5−3_TRBJ2−1
1.5
1.0
0.5
0.0
−2.5
0.0
2.5
5.0
−log10(q value)
log2(ratio of average frequency of VJ usage)
C
VJ usage metastasis vs blood
TRBV7−1_TRBJ2−1
TRBV6−9_TRBJ2−5
TRBV6−9_TRBJ2−3
TRBV7−4_TRBJ2−1
TRBV7−1_TRBJ1−2
TRBV7−1_TRBJ2−5
TRBV6−9_TRBJ1−2
TRBV7−4_TRBJ2−2
TRBV5−3_TRBJ2−3
TRBV6−2_TRBJ2−1
TRBV5−6_TRBJ2−3
TRBV7−1_TRBJ2−3
TRBV7−6_TRBJ2−5
TRBV7−1_TRBJ2−2
TRBV4−1_TRBJ2−1
TRBV7−1_TRBJ1−5
TRBV7−1_TRBJ2−7
TRBV6−9_TRBJ2−6
TRBV6−9_TRBJ1−1
TRBV6−9_TRBJ2−4
TRBV18_TRBJ2−6
TRBV6−7_TRBJ2−5
TRBV7−4_TRBJ1−6
TRBV14_TRBJ2−5
TRBV4−2_TRBJ2−4
TRBV6−2_TRBJ2−5
TRBV7−1_TRBJ1−4
TRBV6−5_TRBJ1−3
TRBV12−4_TRBJ2−1
TRBV7−4_TRBJ2−6
TRBV5−1_TRBJ2−5
TRBV5−3_TRBJ2−4
TRBV14_TRBJ2−4
TRBV7−1_TRBJ1−6
TRBV5−5_TRBJ2−1
TRBV6−9_TRBJ1−4
TRBV7−4_TRBJ2−5
TRBV26_TRBJ2−5
TRBV10−2_TRBJ2−7
TRBV9_TRBJ2−4
TRBV19_TRBJ2−7
TRBV16_TRBJ2−1
TRBV25−1_TRBJ2−4
TRBV9_TRBJ2−3
TRBV6−1_TRBJ2−4
3
2
1
0
−5.0
−2.5
0.0
2.5
5.0
−log10(q value)
log2(ratio of average frequency of VJ usage)
Enrichment
A enriched
B enriched

Supplemental Figure S2 Differential TCR VJ segment usage between blood, normal gastric tissue, primary tumour and metastasis (A-F) VJ usage frequency were averaged across samples in a tissue type. Figure title left of vs represent A enriched, right of vs represent B enriched. Y-axis represent log2 of ratio of those averaged VJ usage frequencies between the two tissue types compared. X-axis represent -log10 of q-value, which is the p-value after multiple testing correction using FDR. Only differentially used VJ segments with q-value <0.2 are labeled.

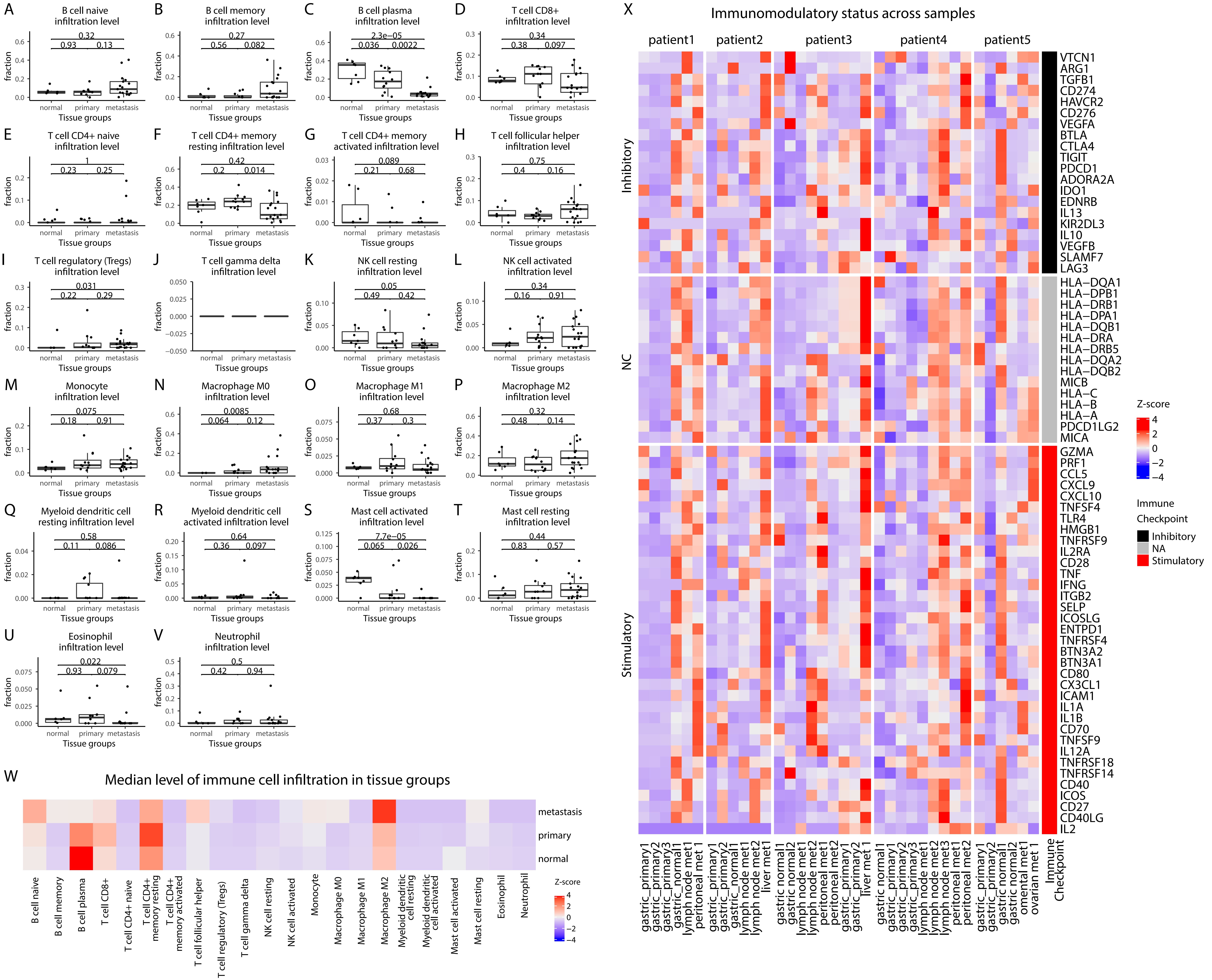

A B cell naive infiltration level
B B cell memory infiltration level
C B cell plasma infiltration level
D T cell CD8+ infiltration level
E T cell CD4+ naive infiltration level
F T cell CD4+ memory resting infiltration level
G T cell CD4+ memory activated infiltration level
H T cell follicular helper infiltration level
I T cell regulatory (Tregs) infiltration level
J T cell gamma delta infiltration level
K NK cell resting infiltration level
L NK cell activated infiltration level
M Monocyte infiltration level
N Macrophage M0 infiltration level
O Macrophage M1 infiltration level
P Macrophage M2 infiltration level
Q Myeloid dendritic cell resting infiltration level
R Myeloid dendritic cell activated infiltration level
S Mast cell activated infiltration level
T Mast cell resting infiltration level
U Eosinophil infiltration level
V Neutrophil infiltration level
fraction
normal primary metastasis
Tissue groups
W Median level of immune cell infiltration in tissue groups
metastasis
primary
normal
Z-score
X Immunomodulatory status across samples
patient1 patient2 patient3 patient4 patient5
Inhibitory
NC
Stimulatory
Immune Checkpoint
Inhibitory
NA
Stimulatory

Supplemental Figure S3 Immune microenvironment status details (A-V) Boxplot of 22 immune cell type infiltration levels between normal, primary and metastatic tissue. Wilcoxon test was used to assess statistical significance. (W) Heatmap of median level of 22 immune cell type infiltration between normal, primary and metastatic tissue. Values are tumour infiltration level (TIL) z-score normalized across each immune cell type. (X) Heatmap of immunomodulatory status across samples, assessed by gene expression levels of 70 genes related to immunomodulation. Values are transcript per million (TPM) gene expression values z-score normalized across each gene, for each patient.

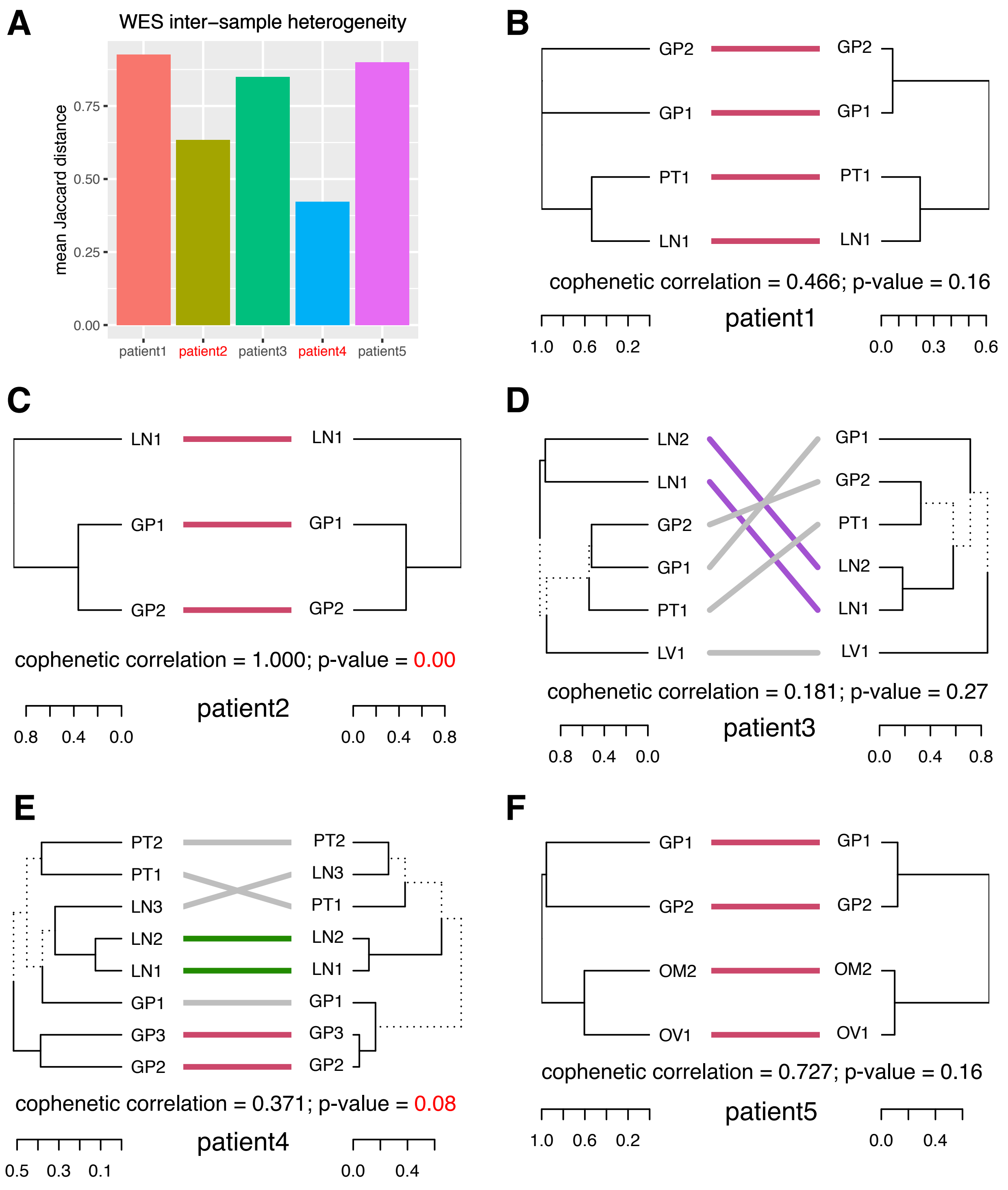

A
WES inter–sample heterogeneity
mean Jaccard distance
0.00
0.25
0.50
0.75
patient1
patient2
patient3
patient4
patient5
B
GP2
GP1
PT1
LN1
cophenetic correlation = 0.466; p-value = 0.16
patient1
1.0 0.6 0.2
0.0 0.3 0.6
C
LN1
GP1
GP2
cophenetic correlation = 1.000; p-value = 0.00
patient2
0.8 0.4 0.0
0.0 0.4 0.8
D
LN2
LN1
GP2
GP1
PT1
LV1
cophenetic correlation = 0.181; p-value = 0.27
patient3
0.8 0.4 0.0
0.0 0.4 0.8
E
PT2
PT1
LN3
LN2
LN1
GP1
GP3
GP2
cophenetic correlation = 0.371; p-value = 0.08
patient4
0.5 0.3 0.1
0.0 0.4
F
GP1
GP2
OM2
OV1
cophenetic correlation = 0.727; p-value = 0.16
patient5
1.0 0.6 0.2
0.0 0.4

Supplemental Figure S4 Mutational heterogeneity among 5 patients and neo-antigen vs TCR repertoire evolution (A) WES mutational inter-sample heterogeneity for a patient was represented by the mean Jaccard distance. Jaccard similarity of mutations of all possible sample pairs in a patient was calculated. 1 – Jaccard similarity equals Jaccard distance. Averaging all Jaccard distances in a patient gives the mean Jaccard distance. (B-F) Neo-antigen and T cell receptor co-evolution. Neo-antigen and TCR trees were constructed using hierarchical clustering in complete mode. Trees were compared using cophenetic correlation.

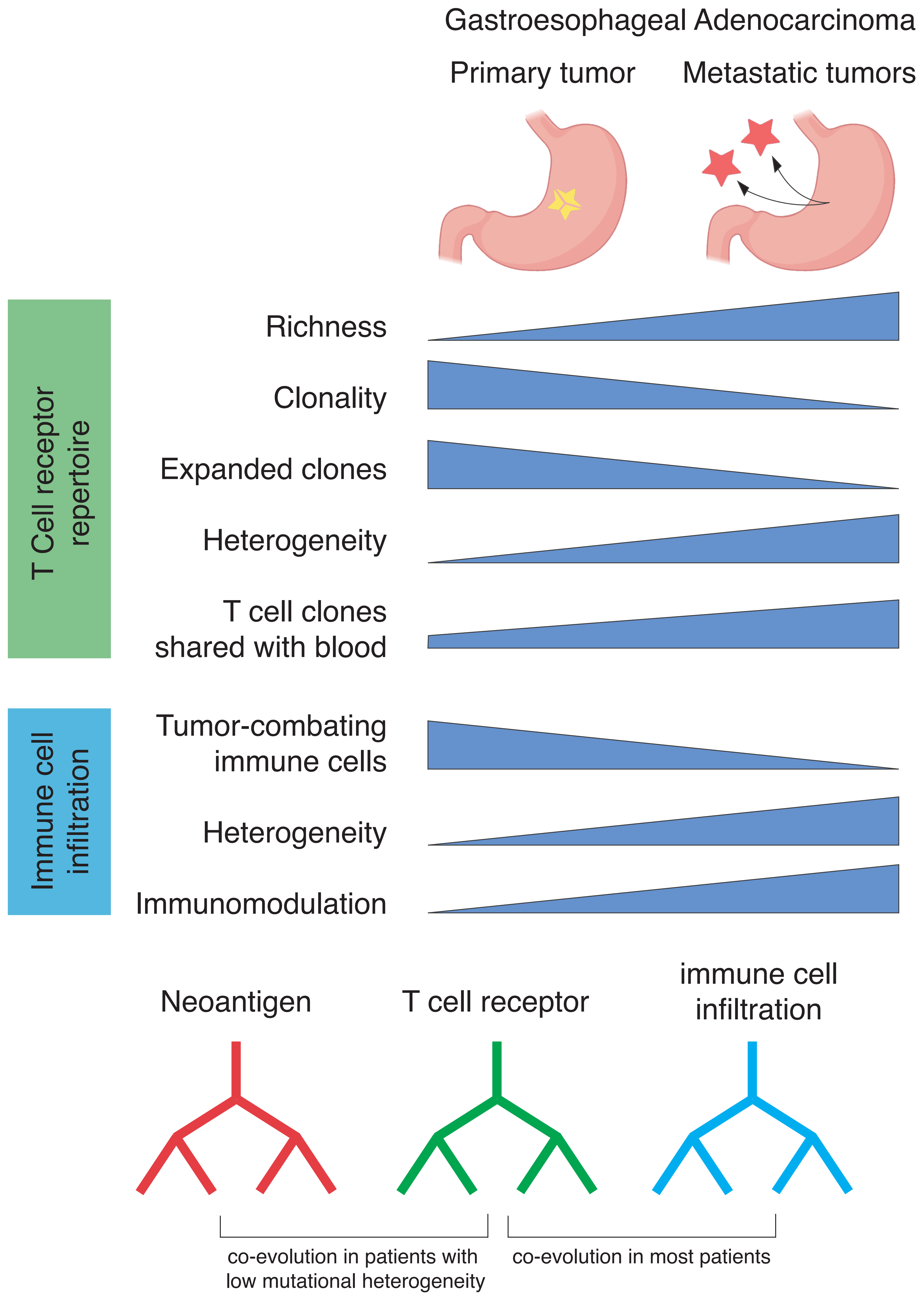
Gastroesophageal Adenocarcinoma
Primary tumor
Metastatic tumors
T Cell receptor repertoire
Richness
Clonality
Expanded clones
Heterogeneity
T cell clones shared with blood
Immune cell infiltration
Tumor-combating immune cells
Heterogeneity
Immunomodulation
Neoantigen
T cell receptor
immune cell infiltration
co-evolution in patients with low mutational heterogeneity
co-evolution in most patients

Supplemental Figure S5 Summary findings of GEA tumour immune microenvironment

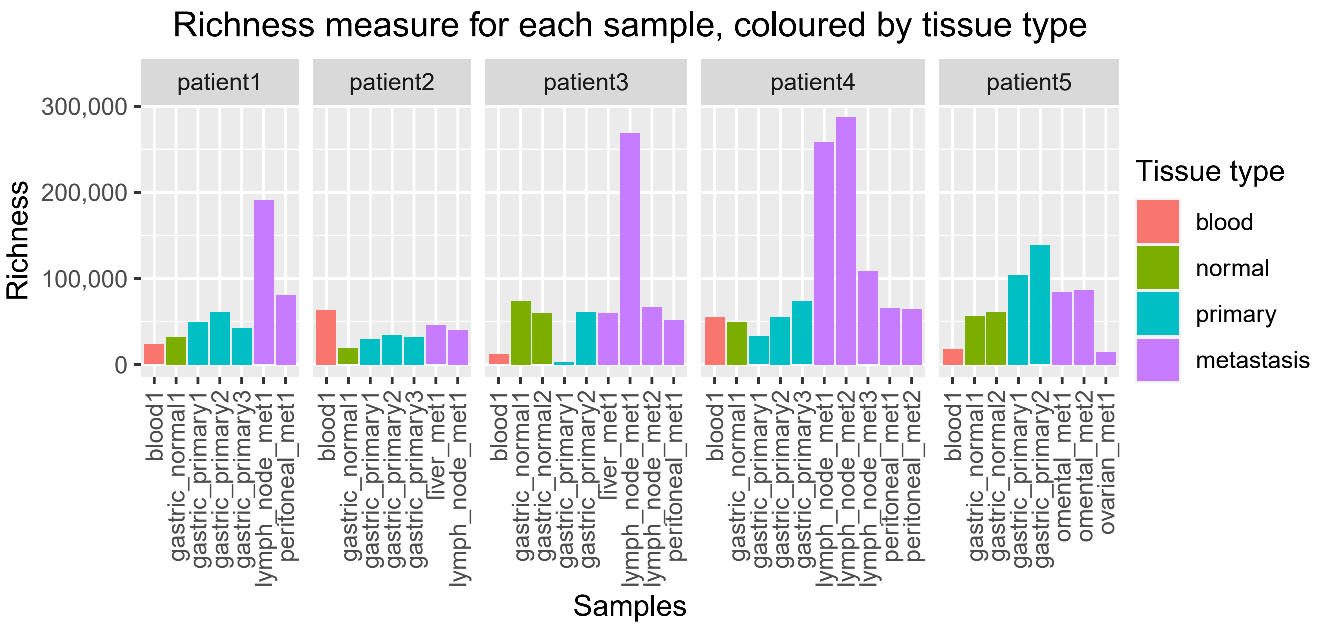

Richness measure for each sample, coloured by tissue type
patient1
patient2
patient3
patient4
patient5
Richness
300,000
200,000
100,000
0
Samples
Tissue type
blood
normal
primary
metastasis
blood1
gastric_normal1
gastric_normal2
gastric_primary1
gastric_primary2
gastric_primary3
lymph_node_met1
lymph_node_met2
lymph_node_met3
peritoneal_met1
peritoneal_met2
liver_met1
omental_met1
omental_met2
ovarian_met1

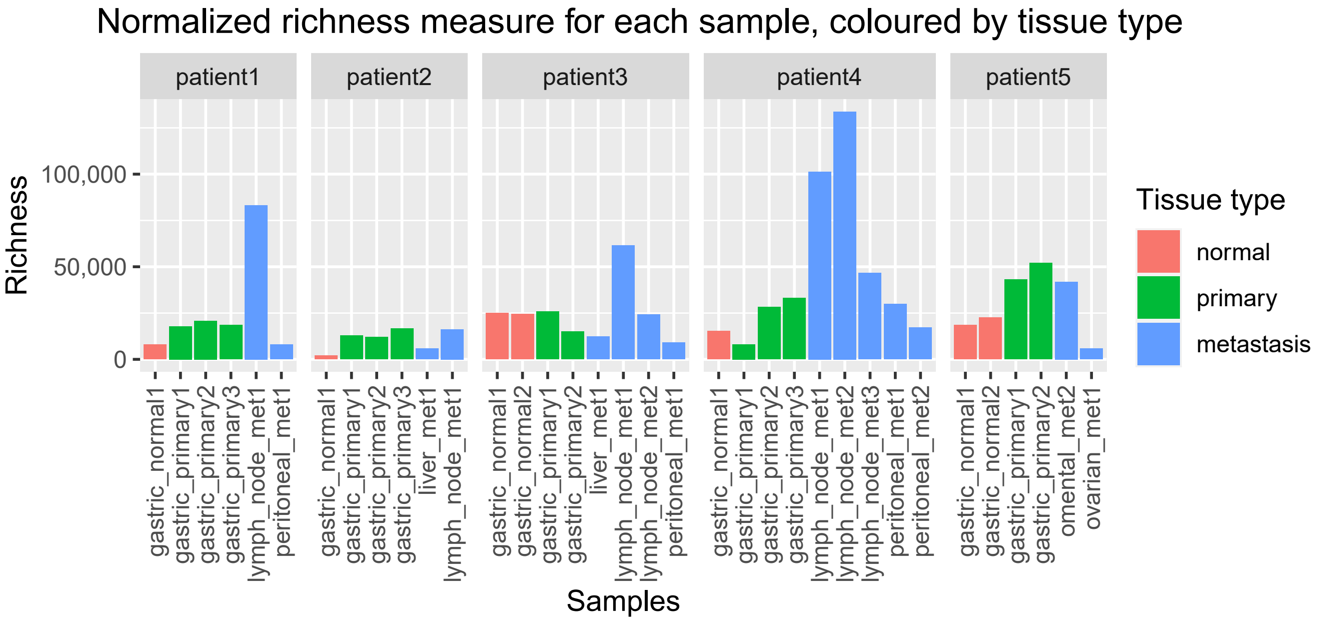

Normalized richness measure for each sample, coloured by tissue type
patient1
patient2
patient3
patient4
patient5
Richness
100,000
50,000
0
Samples
Tissue type
normal
primary
metastasis
gastric_normal1
gastric_normal2
gastric_primary1
gastric_primary2
gastric_primary3
lymph_node_met1
lymph_node_met2
lymph_node_met3
peritoneal_met1
peritoneal_met2
liver_met1
omental_met2
ovarian_met1

Supplemental Figure S6 Comparison of normalized and unnormalized TCR richness. The top bar chart is the same as from Supplementary Figure 2A. The bottom bar chart displays the normalized TCR richness across samples from all patients. Blood samples and samples that did not undergo CIBERSORT were removed from the analysis. Samples are coloured according to their tissue type.

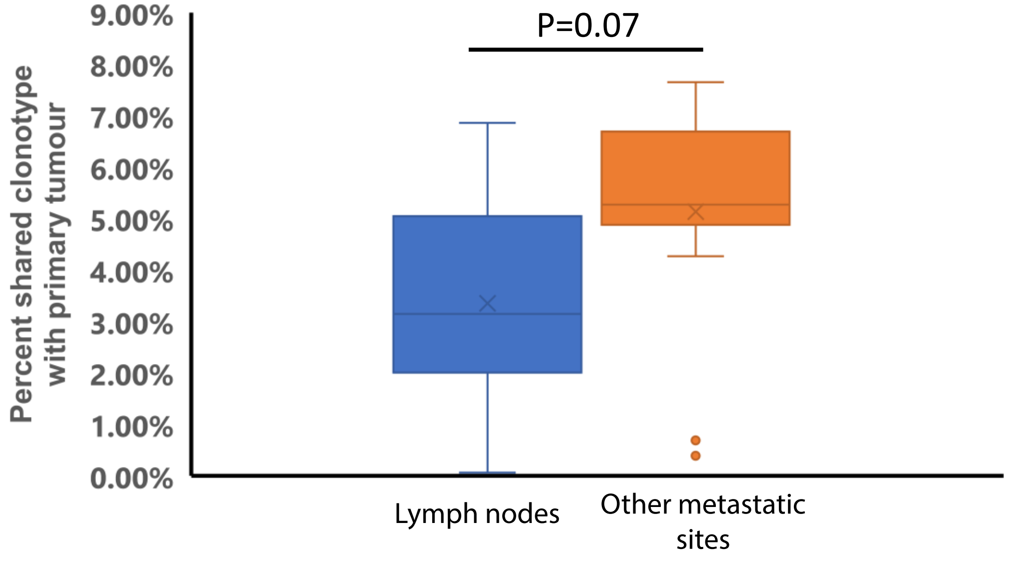

P=0.07
Percent shared clonotype with primary tumour
9.00%
8.00%
7.00%
6.00%
5.00%
4.00%
3.00%
2.00%
1.00%
0.00%
Lymph nodes
Other metastatic sites

Supplemental figure S7 Percent shared TCR clonotype with primary tumour for lymph nodes and other metastatic sites. Two-sided Wilcoxon test was used to determine significance.
